# Computational identification of promising genetic markers associated with molecular mechanisms of reduced rice resistance to Rhizoctonia solani under excess nitrogen fertilization using gene network reconstruction and analysis methods

**DOI:** 10.18699/vjgb-24-103

**Published:** 2024-12

**Authors:** E.A. Antropova, A.R. Volyanskaya, A.V. Adamovskaya, P.S. Demenkov, I.V. Yatsyk, T.V. Ivanisenko, Y.L. Orlov, Ch. Haoyu, M. Chen, V.A. Ivanisenko

**Affiliations:** Institute of Cytology and Genetics of the Siberian Branch of the Russian Academy of Sciences, Novosibirsk, Russia Artificial Intelligence Research Center, Novosibirsk State University, Novosibirsk, Russia; Institute of Cytology and Genetics of the Siberian Branch of the Russian Academy of Sciences, Novosibirsk, Russia Artificial Intelligence Research Center, Novosibirsk State University, Novosibirsk, Russia; Institute of Cytology and Genetics of the Siberian Branch of the Russian Academy of Sciences, Novosibirsk, Russia Artificial Intelligence Research Center, Novosibirsk State University, Novosibirsk, Russia; Institute of Cytology and Genetics of the Siberian Branch of the Russian Academy of Sciences, Novosibirsk, Russia Artificial Intelligence Research Center, Novosibirsk State University, Novosibirsk, Russia Novosibirsk State University, Novosibirsk, Russia Kurchatov Genomic Center of ICG SB RAS, Novosibirsk, Russia; Institute of Cytology and Genetics of the Siberian Branch of the Russian Academy of Sciences, Novosibirsk, Russia Artificial Intelligence Research Center, Novosibirsk State University, Novosibirsk, Russia Kurchatov Genomic Center of ICG SB RAS, Novosibirsk, Russia; Institute of Cytology and Genetics of the Siberian Branch of the Russian Academy of Sciences, Novosibirsk, Russia Artificial Intelligence Research Center, Novosibirsk State University, Novosibirsk, Russia Novosibirsk State University, Novosibirsk, Russia Kurchatov Genomic Center of ICG SB RAS, Novosibirsk, Russia; Institute of Cytology and Genetics of the Siberian Branch of the Russian Academy of Sciences, Novosibirsk, Russia Novosibirsk State University, Novosibirsk, Russia Agrarian and Technological Institute, Peoples’ Friendship University of Russia, Moscow, Russia Digital Health Center, I.M. Sechenov First Moscow State Medical University of the Ministry of Health of the Russian Federation (Sechenovskiy University), Moscow, Russia; Department of Bioinformatics, College of Life Sciences, Zhejiang University, Hangzhou, China; Department of Bioinformatics, College of Life Sciences, Zhejiang University, Hangzhou, China; Institute of Cytology and Genetics of the Siberian Branch of the Russian Academy of Sciences, Novosibirsk, Russia Artificial Intelligence Research Center, Novosibirsk State University, Novosibirsk, Russia Novosibirsk State University, Novosibirsk, Russia Kurchatov Genomic Center of ICG SB RAS, Novosibirsk, Russia

**Keywords:** Oryza sativa, Rhizoctonia solani, plant bioinformatics, differentially expressed genes, genetic regulation, associative gene networks, Smart crop knowledge base, ANDSystem software and information system, nitrogen fertilizer, fungal response, Oryza sativa, Rhizoctonia solani, биоинформатика растений, дифференциально экспрессируемые гены, генетическая регуляция, ассоциативные генные сети, база знаний Smart crop, программно-информационная система ANDSystem, азотные удобрения, ответ на грибную инфекцию

## Abstract

Although nitrogen fertilizers increase rice yield, their excess can impair plant resistance to diseases, particularly sheath blight caused by Rhizoctonia solani. This pathogen can destroy up to 50 % of the crop, but the mechanisms underlying reduced resistance under excess nitrogen remain poorly understood. This study aims to identify potential marker genes to enhance rice resistance to R. solani under excess nitrogen conditions. A comprehensive bioinformatics approach was applied, including differential gene expression analysis, gene network reconstruction, biological process overrepresentation analysis, phylostratigraphic analysis, and non-coding RNA co-expression analysis. The Smart crop cognitive system, ANDSystem, the ncPlantDB database, and other bioinformatics resources were used. Analysis of the molecular genetic interaction network revealed three potential mechanisms explaining reduced resistance of rice to R. solani under excess nitrogen: the OsGSK2-mediated pathway, the OsMYB44-OsWRKY6-OsPR1 pathway, and the SOG1-Rad51-PR1/PR2 pathway. Potential markers for breeding were identified: 7 genes controlling rice responses to various stresses and 11 genes modulating the immune system. Special attention was given to key participants in regulatory pathways under excess nitrogen conditions. Non-coding RNA analysis revealed 30 miRNAs targeting genes of the reconstructed gene network. For two miRNAs (Osa-miR396 and Osa-miR7695), about 7,400 unique long non-coding RNAs (lncRNAs) with various co-expression indices were found. The top 50 lncRNAs with the highest co-expression index for each miRNA were highlighted, opening new perspectives for studying regulatory mechanisms of rice resistance to pathogens. The results provide a theoretical basis for experimental work on creating new rice varieties with increased pathogen resistance under excessive nitrogen nutrition. This study opens prospects for developing innovative strategies in rice breeding aimed at optimizing the balance between yield and disease resistance in modern agrotechnical conditions.

## Abstract (ru)

Азотные удобрения, повышающие урожайность риса, при избытке могут снижать устойчивость растений к заболеваниям, в частности к ризоктониозу, вызываемому Rhizoctonia solani. Этот патоген способен уничтожить до 50 % урожая, однако механизмы, лежащие в основе снижения устойчивости при избытке азота, остаются малоизученными. Данное исследование направлено на выявление потенциальных генов-маркеров для повышения устойчивости риса к R. solani в условиях избытка азота. Применен комплексный биоинформатический подход, включающий анализ дифференциальной экспрессии генов, реконструкцию генных сетей, анализ перепредставленности биологических процессов, филостратиграфический анализ и анализ коэкспрессии некодирующих РНК. Использованы когнитивная система Smart crop, ANDSystem, база данных ncPlantDB и другие биоинформатические ресурсы. Анализ молекулярно-генетической сети взаимодействий выявил три потенциальных механизма, объясняющих снижение устойчивости риса к R. solani при избытке азота: OsGSK2-опосредованный путь, путь OsMYB44-OsWRKY6-OsPR1 и путь SOG1-Rad51-PR1/PR2. Идентифицированы потенциальные маркеры для селекции: 7 генов, контролирующих ответы риса на широкий круг стрессов, и 11 генов-модуляторов иммунной системы. Особое внимание уделено ключевым участникам регуляторных путей в условиях избытка азота. Анализ некодирующих РНК выявил 30 микроРНК, мишенями которых являются гены из реконструированной генной сети. Для двух микроРНК (Osa-miR396 и Osa-miR7695) обнаружено около 7400 тыс. уникальных длинных некодирующих РНК (днРНК) с различными индексами коэкспрессии. Выделены топ-50 днРНК с наибольшим индексом коэкспрессии для каждой микроРНК, что открывает новые перспективы в изучении регуляторных механизмов устойчивости риса к патогенам. Полученные результаты создают теоретическую основу для экспериментальных работ по созданию новых сортов риса с повышенной устойчивостью к патогенам в условиях избыточного азотного питания.

## Introduction

Rice (Oryza sativa L.) is one of the most economically valuable crops in the world, constituting the main part of the diet for about half of the world’s population. Nitrogen fertilizers are widely used in rice production in agricultural enterprises. They account for about 80–90 % of the yield increase obtained from mineral fertilizers (Kumeiko et al., 2013). However, along with the positive effect, nitrogen fertilizers reduce rice resistance to diseases. Excess nitrogen fertilization is one of the main factors contributing to the development of sheath blight disease in rice, caused by the fungus Rhizoctonia solani Kühn. Sheath blight causes serious damage to this crop’s yield, leading to losses of up to 50 % (Senapati et al., 2022).

Plant susceptibility to pathogenic infections under excess nitrogen fertilization is caused by a complex of factors related to both rapid growth and development, as well as changes in plant defense responses. Excess nitrogen leads to a series of physiological changes that can increase plant susceptibility to pathogens. In particular, accelerated growth can cause weakening of cellular structures, including reduced cell wall strength and decreased cuticle thickness, which facilitates pathogen penetration (Hückelhoven, 2007; Rose et al., 2018). Furthermore, excessive nitrogen nutrition can cause changes in the plant microbiome and stimulate the growth of pathogenic microorganisms in the rhizosphere (Xiong et al., 2021).

At the molecular genetic level, complex regulatory networks including phytohormones, transcription factors, and non-coding RNAs play a key role in forming pathogen resistance. These components participate in complex stress response mechanisms affecting plant immune processes

Phytohormones, such as salicylic acid, brassinosteroids, jasmonic acid, gibberellins, abscisic acid, auxins, and ethylene, have special significance in response to pathogenic infections (Yang J. et al., 2019). Notably, some of these phytohormones, particularly salicylic and abscisic acids, are also involved in nitrogen compound metabolism, regulating the expression of genes related to nitrogen exchange (Xing et al., 2023). This observation suggests that interference in phytohormone signaling pathways may serve as a mechanism through which excess nitrogen affects plant resistance to pathogens

Non-coding RNAs (ncRNAs) represent a diverse group of RNA molecules that are not translated into proteins but perform important regulatory functions in the cell. Among them, several main types are distinguished: microRNAs (miRNA), small interfering RNAs (siRNAs), piRNAs (Piwi-interacting RNAs), ribosomal RNAs (rRNAs), transfer RNAs (tRNAs), and long non-coding RNAs (lncRNAs). Long non-coding RNAs are of particular interest as they play a significant role in gene regulation, affecting mRNA stability and translation, and participating in signaling pathways. In particular, the work of Supriya et al. (2024) shows that lncRNAs are involved in rice response to the fungus R. solani.

Despite their importance, lncRNAs remain the least studied among non-coding RNAs (Statello et al., 2021). This is due to their diversity, complexity of functions and mechanisms of action, as well as technical difficulties in their identification and characterization. One approach to studying the functional role of non-coding RNAs is to analyze their co-expression with protein-coding genes, as well as with other types of non-coding RNAs, the function of which has been established. The most comprehensive resource for non-coding RNA co-expression, including rice long non-coding RNAs, is the ncPlantDB database (https://bis.zju.edu.cn/ncPlantDB/).

The study of interactions between these various regulatory elements – phytohormones, transcription factors, and noncoding RNAs – in the context of nitrogen metabolism and pathogen resistance represents a promising research direction. It may lead to a deeper understanding of the mechanisms underlying nitrogen-induced plant disease susceptibility and potentially reveal new ways to enhance crop resistance under intensive nitrogen nutrition

A widely used approach in computational systems biology for studying complex molecular genetic processes is the gene network method (Kolchanov et al., 2013). For automatic reconstruction of gene networks, the Institute of Cytology and Genetics of SB RAS has developed the ANDSystem cognitive system, which uses artificial intelligence methods to extract knowledge from databases and scientific publication texts (Ivanisenko V.A. et al., 2015, 2019). ANDSystem has been successfully applied to reconstruct associative gene networks and interpret genomic, proteomic, and metabolomic data in various fields of biomedicine and agrobiology. In particular, this software system has been used to reconstruct important molecular genetic mechanisms of various pathological processes and biological phenomena, including asthma (Bragina et al., 2014; Saik et al., 2018; Zolotareva et al., 2019), lymphedema (Saik et al., 2019), tuberculosis (Bragina et al., 2016), hepatitis C (Saik et al., 2016), coronavirus infection (Ivanisenko V.A. et al., 2022), Huntington’s disease (Bragina et al., 2023), glioma (Rogachev et al., 2021), post-operative delirium (Ivanisenko V.A. et al., 2023), and others.

In the field of plant biology, ANDSystem has enabled new discoveries about the molecular mechanisms of cell wall functioning in Arabidopsis thaliana L. leaves in response to drought (Volyanskaya et al., 2023). Adapting ANDSystem’s knowledge extraction methods to potato biology led to the creation of the specialized SOLANUM TUBEROSUM knowledge base, containing information about genetic regulation of potato metabolic pathways (Ivanisenko T.V. et al., 2018), which was used to prioritize potato genes involved in the formation of agronomically valuable traits (Demenkov et al., 2019).

The aim of this study was to conduct a comprehensive bioinformatic analysis of molecular mechanisms of rice response to R. solani under excess nitrogen conditions. The study included gene network reconstruction using the Smart Crop knowledge base – a specialized version of ANDSystem configured for rice biology, as well as the application of bioinformatic methods for analyzing the overrepresentation of biological processes, phylostratigraphic analysis of gene evolutionary age, and analysis of non-coding RNA co-expression.

## Materials and methods

**Fig. 1.**
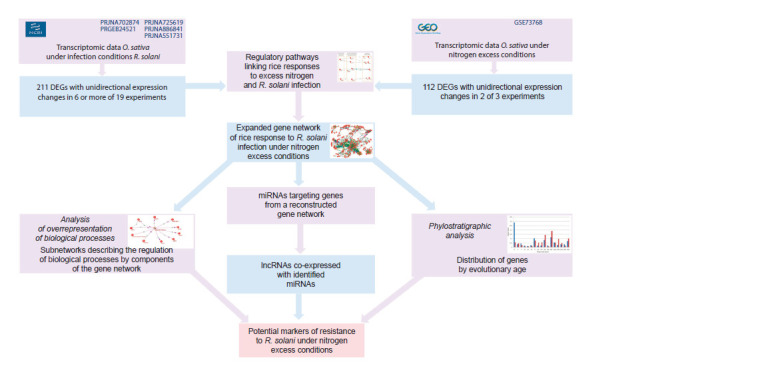
Research stage diagram.

The study was conducted in several sequential stages (Fig. 1). In the first stage, based on transcriptome data analysis, genes that had been differentially expressed during R. solani infection were identified, as well as genes, the differential expression of which had been observed under excess nitrogen conditions. The second stage included the reconstruction of regulatory gene networks involving the identified genes. In the third stage, a structural and functional analysis of the obtained networks was conducted, including assessment of node centrality measures, analysis of biological process enrichment, and determination of gene evolutionary age. Next, analysis of network gene translation regulation by miRNAs was performed, and long non-coding RNA co-expression was investigated. The final stage was aimed at identifying potential markers of resistance to R. solani under excess nitrogen conditions.

**Publicly available gene expression data.** Publicly available transcriptomic data on O. sativa response to excess nitrogen fertilization, as well as to the pathogen R. solani, were collected from the NCBI GEO (Gene Expression Omnibus) and NCBI SRA (Sequence Read Archive) databases (https:// www.ncbi.nlm.nih.gov/sra) (Table 1). For the analysis of O. sativa transcriptome under excess nitrogen conditions, one study containing three experiments was found. In this work, plants were treated with excess fertilizer – ammonium nitrate (NH4NO3) – at concentrations exceeding the normal level by 4, 16, and 64 times.

**Table 1.**
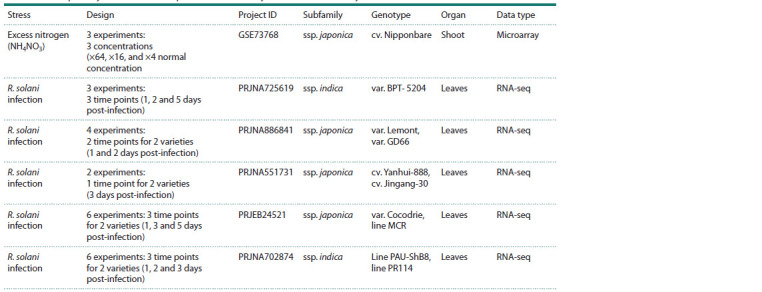
List of publicly available RNA-seq and DNA microarray data used in the study

The differential expression analysis of O. sativa during R. solani infection included data from five time-series studies, containing a total of 21 experiments.

** Transcriptomic data analysis.** SRA Toolkit (v3.1.0) was used to extract FASTQ format files. Read quality control was performed using FastQC (v0.12.0). Filtering and removal of low-quality nucleotides was conducted using Trimmomatic (https://github.com/usadellab/Trimmomatic). A read length of 15 bp and Phred sequence quality score < Q20 were used as thresholds. Reads were mapped to the reference genome of O. sativa Japonica Group (IRGSP-1.0), deposited from the EnsemblPlants database (https://plants.ensembl.org/index.html) using the HISAT2 (v2.2.1) tool. SAMtools (v1.20) was used to convert SAM format mapping output files to binary BAM format. HTSeq (v2.0.2) was used for quantification. Read count normalization and differential gene expression analysis were performed using the edgeR (4.0.16) tool implemented in the Bioconductor project (https://www.bioconductor.org/). The TMM (Trimmed Mean of M-values) method was used for normalization. Multiple testing correction was applied using FDR (false discovery rate).

For DNA microarray data analysis, the limma (v3.58.1) package from the Bioconductor project was used. Raw Agilent platform DNA microarray files were read using read.images. Background noise correction and quantile normalization of the data were then performed. The biomaRt (v2.58.2) package (https://bioconductor.org/packages/release/bioc/html/ biomaRt.html) was used to map DNA microarray probe identifiers to Ensembl gene identifiers. Differential gene expression analysis was performed using the limma package. An FDR threshold of < 0.05 was used to identify differentially expressed genes.

Smart Crop knowledge base. This study used the specialized Smart Crop knowledge base, which is an adapted version of the ANDSystem software and information system, focused on rice and wheat genetics and breeding. System adaptation included configuring three key ANDSystem modules for effective task solving. The first module was the domain-specific ontology module, which was expanded with special dictionaries. These dictionaries covered a wide range of research objects that can be divided into molecular genetic objects (genes, proteins, metabolites, non-coding RNAs, and miRNAs), their functional characteristics (biological processes, genetic biomarkers, QTL polymorphisms), phenotypic characteristics (plant varieties, breeding-significant qualities, phenotypic traits, diseases), biotic and abiotic factors (pathogens, pests, and others). Various databases and ontologies were used to form these dictionaries, such as NCBI Gene (https://www.ncbi. nlm.nih.gov/gene/), ChEBI (https://www.ebi.ac.uk/chebi/), MirBase (https://www.mirbase.org/), Gene Ontology (https:// cropontology.org/), Wheat Ontology, Rice Ontology, and others (Chao et al., 2023). For example, the gene dictionary from the molecular genetic objects group contains names of approximately 627 thousand genes, including their conventional names and synonyms. Biological processes, belonging to functional characteristics, contain more than 122 thousand names. The pathogen dictionary, included in the biotic factors group, contains about 755 names.

The second important component was the information extraction module from factographic databases, which was configured for automated data extraction from specialized sources in plant biology. These sources included Oryzabase (https://shigen.nig.ac.jp/rice/oryzabase/), GrainGenes (https:// wheat.pw.usda.gov/GG3/), ASPNet, and others. The third module was the text-mining module using semantic-linguistic templates and artificial intelligence methods. It was adapted for effective knowledge extraction from text sources, such as scientific articles and patents in plant biology. Based on the analysis of scientific publications performed using this module, more than 4 million interactions between objects represented in the dictionaries were extracted.

**Fig. 2.**
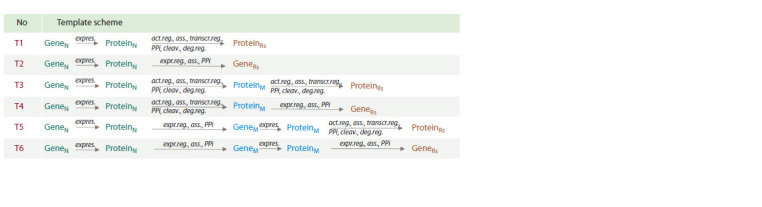
Template scheme used for searching for molecular genetic pathways in the Smart Crop knowledge base. Notation: T – template; GeneN – DEGs of rice under excess nitrogen fertilization; ProteinN – protein products of DEGs under excess nitrogen fertilization; GeneM – genes encoding mediator proteins; ProteinM – mediator proteins; GeneRs – rice DEGs in response to R. solani; ProteinRs – protein products of rice DEGs in response to R. solani; expres. – expression; act.reg. – regulation of activity; expr.reg. – regulation of expression; ass. – association; transcr.reg. – regulation of transcription; deg.reg. – regulation of degradation; cleav. – cleavage; PPi – protein-protein interaction.

**Gene network reconstruction and analysis.** Gene network reconstruction was performed using the “Query Wizard” and “Pathway Wizard” of the ANDVisio software module (Demenkov et al., 2011), which serves as the user interface in the ANDSystem and Smart Crop systems. The structure of templates used for searching for regulatory pathways in the Smart Crop knowledge base using the “Pathway Wizard” is shown in Figure 2.

**Node centrality assessment in the gene network.** Node centrality in the gene network was evaluated using the network connectivity measure, defined as the number of connections between a given node and other network nodes.

**Biological process enrichment analysis.** Gene Ontology biological process enrichment analysis was performed using the PANTHER resource (https://pantherdb.org/).

**Long non-coding RNA analysis.** Co-expression analysis between miRNAs and lncRNAs was conducted using the ncPlantDB database (https://bis.zju.edu.cn/ncPlantDB/).

** Phylostratigraphic analysis. **The evolutionary age of genes was determined using the GenOrigin database (http://chenzxlab.hzau.edu.cn/) (Tong et al., 2021), which contains information about the evolutionary age of genes from various organisms, established through phylostratigraphic analysis. To assess the statistical significance of differences in the distribution of genes of different ages between the complete set of rice protein-coding genes and genes in the reconstructed network, a hypergeometric test was applied. The probability of observing m or more genes of a certain age interval among M network genes was calculated using the hypergeom.pmf function from the scipy library. The analysis was conducted for 17 age intervals represented in the GenOrigin database. The following parameters were used in calculations: N – total number of rice protein-coding genes, n – number of rice genes in a given age interval, M – number of genes in the gene network, m – number of network genes in the analyzed age interval. Differences were considered statistically significant at p-value < 0.05.

## Results and discussion

Identification of stable differentially expressed genes

To identify differentially expressed genes (DEGs) in rice under excess nitrogen conditions, 3 experiments were analyzed, while under R. solani fungus influence, 21 experiments were analyzed using transcriptomic data found in open sources. We considered genes with unidirectional expression changes across different experiments (simultaneous decrease or increase), which we will further refer to as stable DEGs.

In the case of excess nitrogen, only 5 genes were found to be stable DEGs across all three experiments (Os09g0538000, Os05g0162000, Os09g0537700, Os04g0664900, Os06g0113800). When considering DEGs present in two out of three experiments, the number of such genes was 112, which were taken for further analysis

Analysis of differential gene expression under R. solani infection showed that in two out of 21 experiments, no statistically significant DEGs were identified. Analysis of the remaining 19 experiments revealed no genes that were DEGs in every experiment. Only 2 genes were found to be stable DEGs in half or more of the experiments (Os04g0180500 and Os09g0255600). When considering one-third of the experiments (6 or more out of 19), the number of stable DEGs included 211 genes. The number of stable DEGs for a quarter of the experiments (5 or more out of 19) was 463 genes. For further analysis, we chose a threshold value for determining stable DEGs equal to one-third of the experiments (6 or more out of 19), as at this value, the samples of stable DEGs under excess nitrogen and fungal influence were comparable in size.

Reconstruction of molecular genetic pathways describing the relationship between rice responses to excess nitrogen and infection

**Fig. 3.**
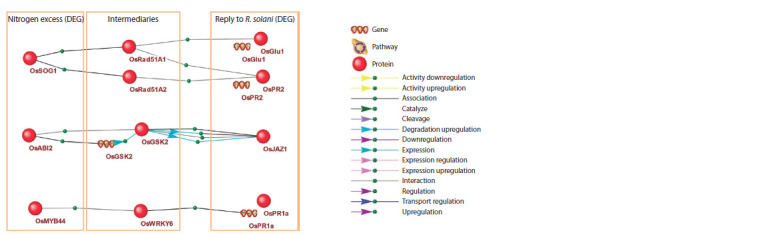
Regulatory pathways describing the connection between DEGs in rice response to excess nitrogen and R. solani infection.

Using the ANDVisio program, which serves as the user interface for the Smart Crop and ANDSystem knowledge bases, a search was conducted for molecular genetic pathways in the global Smart Crop gene network (Fig. 2), connecting the group of the selected 112 stable DEGs in response to excess nitrogen and 211 stable DEGs in response to R. solani fungus. This search resulted in the identification of several regulatory pathways that included 3 proteins encoded by DEGs in response to excess nitrogen, 4 DEGs and their encoded proteins in response to R. solani infection, as well as 4 proteins acting as mediators in interactions between the considered DEGs (Fig. 3).

OsABI2-OsGSK2-OsJAZ1 molecular genetic pathway

An important reconstructed pathway (Fig. 3) potentially explaining the mechanism of deteriorated rice resistance to fungus under excess nitrogen is the OsABI2-OsGSK2- OsJAZ1 pathway. The OsABI2 protein (PP2C06, protein phosphatase 2C6) is a product of the Os01g0583100 gene that is differentially expressed under excess nitrogen: its expression decreases at 16- and 64-fold excess of nitrogen fertilizer concentration (Supplementary Material 1)1.


Supplementary Materials are available in the online version of the paper: https://vavilov.elpub.ru/jour/Suppl_Antropova_Engl_28_8.xlsx


It is known that ABI2 is one of the main participants in the ABA (abscisic acid) signaling pathway (Sun et al., 2011), which is an important plant hormone necessary for regulating stomatal closure, leaf senescence, bud dormancy, seed germination inhibition, growth inhibition, and stress responses to drought, salinity, and toxic metals (Chen et al., 2020; Kumar S. et al., 2022). Literature has shown that OsABI2 participates in rice response to excess iron (Junior et al., 2015), in sunflower, its expression increases during drought (Shen et al., 2023), and in rice, during drought, its expression is also noted in roots and stem (Sircar et al., 2022). The presence of this protein in the reconstructed regulatory pathway may indicate its involvement in modulating rice response to the pathogen under excess nitrogen. OsABI2 can exert regulatory influence on OsJAZ1 (jasmonate-Zim-domain protein 1), an important factor in pathogen response, through the mediator OsGSK2.

According to our analysis, OsJAZ1 (Os10g0392400) is a DEG with increased expression levels in 7 out of 19 experiments studying R. solani influence on rice transcriptome (Supplementary Material 2). In Arabidopsis and cotton, it was shown that the fungus Verticillium dahliae, which causes Verticillium wilt, induces JAZ1 phosphorylation through GSK2, and this promotes further JAZ1 degradation (Song Y. et al., 2021). The authors note that in this action, GSK2 is a negative regulator of fungal resistance – its constitutive expression weakened resistance, while GSK2 gene knockdown increased resistance to V. dahliae. Interestingly, OsGSK2 (Os05g0207500) is a DEG in 2 out of 19 analyzed experiments studying R. solani influence on transcriptome, where its expression was decreased (Supplementary Material 2). Also, OsGSK2 is a DEG in response to excess nitrogen in the experiment with the highest nitrogen fertilizer concentration (64 times higher than normal concentration).

In our network, the connection between ABI2 and GSK2 is of the “interaction” type (physical interaction). In Arabidopsis, it was shown that ABI1 and ABI2 interact with the GSK2 protein (Glycogen synthase kinase 2, also known as: brassinosteroid insensitive 2, BIN2) and dephosphorylate it, leading to suppression of its kinase activity and decreased stability. The examined interactions between regulatory pathway participants are consistent with literature data showing that the abscisic acid signaling pathway suppresses the brassinosteroid signaling pathway (Wang H., 2018). In particular, in O. sativa, it was demonstrated that ABA acts oppositely to BR (brassinosteroids) in regulating leaf inclination through the BR biosynthesis gene OsD11 and signaling genes OsGSK2 and OsDLT (Li et al., 2019).

It should be noted that BR represents an important group of plant hormones, in some cases playing an antagonistic role to ABA action. For example, it was shown that BR stimulates seed germination, while ABA promotes their dormancy (Steber, McCourt, 2001).

MYB44-WRKY6-PR1 molecular genetic pathway

Another important regulatory pathway begins with the OsMYB44 protein – a product of the Os09g0106700 gene that is differentially expressed under excess nitrogen. Notably, it is a DEG in two out of three experiments (gene expression is decreased at 16- and 64-fold excess of nitrogen fertilizer concentration, Supplementary Material 1). The transcription factor MYB44 is known to be an important participant in plant life regulation (root development, somatic embryogenesis, leaf senescence, etc.) and response to biotic and abiotic stresses, such as reactions to drought, cold, phosphate and nitrogen deficiency, and pathogenic organism infection (Wang F. et al., 2023). Interestingly, MYB44 has opposing effects on plant defense reactions. Shim et al. (2013) showed that it enhanced the defensive response to pathogenic bacteria Pseudomonas syringae pv. tomato induced by salicylic acid but reduced the defensive response against the black spot disease fungus Alternaria brassicicola, which is dependent on jasmonic acid. In the pathway under consideration, MYB44 forms a regulatory complex with another TF, WRKY6 (Os03g0798500), which regulates inorganic phosphate transport, as shown in potato (Zhou et al., 2017). The transcription factor WRKY6, like MYB44 in A. thaliana, acts as a positive regulator of abscisic acid signaling. The WRKY TF family participates in protecting plants from a wide range of stresses, in particular, OsWRKY6 is necessary for rice protection from Xanthomonas oryzae pv. oryzae (bacterial leaf blight) (Im et al., 2024). It has been shown that OsWRKY6 activates OsPR1 expression (Im et al., 2022), the final link in the regulatory pathway under consideration. SOG1-Rad51-PR1/PR infections (Ogita et al., 2018; Yoshiyama, Kimura, 2018). According to our transcriptional data analysis, SOG1 (Os06g0267500) is a DEG under excess nitrogen (expression level increases in two out of three experiments – at 16- and 64-fold excess of nitrogen fertilizer concentration, Supplementary Material 1).

SOG1 is known to be a transcriptional regulator of OsRad51 (Ogita et al., 2018; Yoshiyama, Kimura, 2018), acting as a mediator in the pathway under consideration. RAD51 is a regulatory protein of plant immune response, and among its direct targets are members of the pathogenesis-related protein family, such as PR1 and PR2 (Wang S. et al., 2010). These genes were among the DEGs in response to R. solani fungus (Supplementary Material 2).

PR1 (Os07g0129200) expression increased in 6 out of 19 experiments studying R. solani influence on transcriptome. Seven genes named PR2 have been found in the rice genome (Yokotani et al., 2014). According to our data, expression of three of them (Os07g0539900, Os01g0940700, and Os01g0940800) increased in 7 out of 19 experiments

It should be noted that the PR1 and PR2 genes were also among the DEGs based on our analysis of transcriptomic data from a series of experiments studying excess nitrogen. Their expression changed significantly in one out of three experiments, where the concentration of nitrogen fertilizers was maximal.

Reconstruction of extended gene network of rice response to R. solani infection under excess nitrogen

**Fig. 4.**
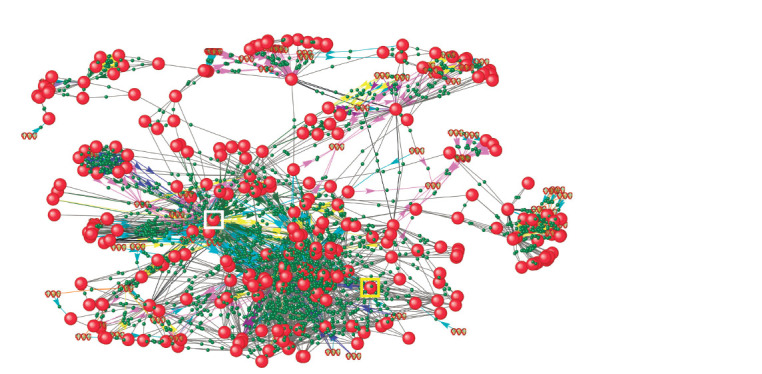
Extended gene network of rice response to R. solani infection under excess nitrogen conditions The network includes both initial regulatory pathways and newly identified components (genes and proteins). The JAZ1 and GSK2 proteins are highlighted with yellow and white squares, respectively. Gene and protein designations and their interaction types are similar to those shown in Figure 3.

To identify a broader range of potential participants in the mechanisms of deteriorating rice resistance to R. solani fungus under excess nitrogen, we reconstructed an extended gene network based on the regulatory pathways discussed above. Gene network reconstruction was performed automatically using the functional module of the ANDVisio program. This tool allows expanding the initial network by adding new components (genes, proteins, metabolites, etc.) based on data about their interactions contained in the Smart Crop knowledge base. For 15 participants of the initial regulatory pathways (Fig. 3), the knowledge base contained information about their interactions with 358 new proteins and genes. The network reconstructed in this way contained 61 genes, 271 proteins, and 2,359 interactions (Fig. 4). To identify key participants in the reconstructed network, node centrality analysis was conducted using the “Network connectivity” index, indicating the number of nearest neighbors. The highest index value belonged to the OsGSK2 protein, which is a participant in the initial regulatory pathways, mediating interactions between differentially expressed genes. Jaz1 was also among the top three in terms of the “Network connectivity” index. It should be noted that the gene encoding Jaz1 was a stable DEG in response to R. solani fungus.

Identification of lncRNAs potentially regulating the identified molecular genetic pathways

To search for lncRNAs potentially involved in regulating the rice gene network response to fungus under excess nitrogen conditions, we analyzed the ncPlantDB database. This database contains information about lncRNA co-expression with miRNAs, obtained from single-cell data analysis.

According to the Smart Crop knowledge base, we found 30 miRNAs that target genes from the reconstructed gene network (Table 2). In the ncPlantDB database, co-expression connections were found for Osa-miR396 and Osa-miR7695 with lncRNAs, with various co-expression degree indices. For two variants of Osa-miR396 (Osa-miR396b and OsamiR396c), the number of such non-coding RNAs was around 4,000. For Osa-miR7695, about 3,500 co-expression connections with lncRNAs were identified. The total number of unique lncRNAs was approximately 7,400.

**Table 2.**
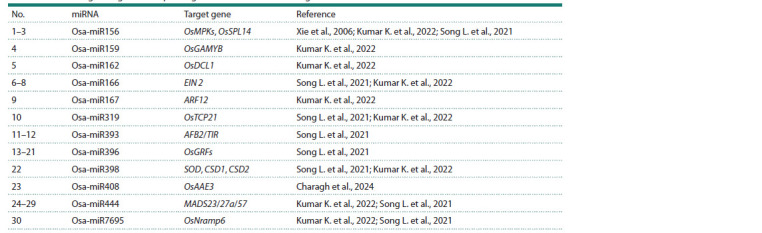
miRNAs regulating stress response genes in the reconstructed gene network Note. miRNAs of the same family are grouped together.

Among the identified lncRNAs, special attention should be paid to those with the highest co-expression index. These include the top 50 lncRNAs ranked by co-expression index, particularly the group of lncRNAs identified in rice metaxylem that have the same co-expression index with Osa-miR396b, the target genes of which are GFR1 and GFR3: LNC-Os08g15450, LNC-Os04g61735, LNC-Os05g27975, LNC-Os05g62500, and others (Supplementary Material 3).

The search for functions of these lncRNAs in literature data yielded no results. Therefore, the connection of lncRNAs with the gene network may have special significance for further characterization of their functions

Phylostratigraphic analysis

The application of phylostratigraphic analysis methods to assess the evolutionary age of genes is a promising approach to studying the evolution patterns of gene networks and identifying their key components (Mustafin et al., 2021). In this work, this approach was used to analyze the evolutionary stages at which genes participating in the reconstructed network of response to fungal infection under elevated nitrogen fertilizer concentrations emerged.

**Fig. 5.**
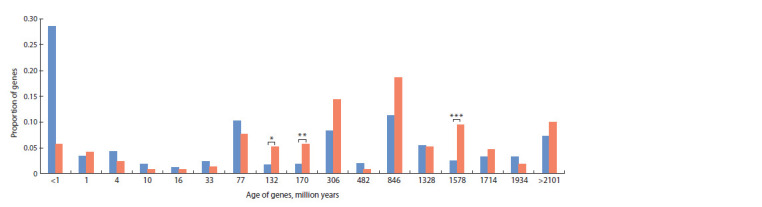
Distribution of gene evolutionary age in the reconstructed gene network The X-axis shows the central points of age intervals (million years) according to the GenOrigin database, the Y-axis shows the proportion of genes in each age interval. Blue shows the distribution for the complete set of rice protein-coding genes, red shows the distribution for genes in the reconstructed network. Asterisks mark age intervals with statistically significant differences in gene representation: * p = 1.85 · 10–3, ** p = 9.16 · 10–4, *** p = 5.41 · 10–7, hypergeometric test.

Analysis of the evolutionary age distribution of genes showed that the reconstructed network contains genes of different ages, among which several most represented groups can be distinguished (Fig. 5). Age intervals within which the number of genes statistically significantly exceeded the one expected by chance corresponded to the following time points shown in the graph (Fig. 5): (1) 132 million years (p = 1.85·10–3), (2) 170 million years (p = 9.16·10–4), and (3) 1,578 million years (p = 5.41·10–7).

The first group, including 11 genes about 132 million years old, likely emerged at the evolutionary stage of monocot plant appearance (Friis et al., 2004). Representatives of this group include the transcription factor OFP3 (ovate family protein 3). The OFP family is plant-specific, participating in regulation of cellular pluripotency, morphogenesis, and growth in A. thaliana (Wang F. et al., 2016). Moreover, it is suggested that changes in transcription factor regulatory networks are an essential feature of monocot plant evolution (Vincentz et al., 2004).

Within the second interval under consideration (170 million years), the age of 12 genes was found. This period is associated with the emergence of flowering plants (van der Kooi, Ollerton, 2020). Members of the WRKY transcription factor family (WRKY6, 40, and 46), involved in molecular mechanisms of flowering regulation (Song H. et al., 2024), fell into this interval. Importantly, WRKY6 is also a participant in the initial regulatory pathways.

The third group included 20 genes, the age of which fell within the third interval (1,578 million years), corresponding to the emergence of red and green algae (Zhang S. et al., 2021). One representative of this group is the PHT1 (PHOSPHATE TRANSPORTER1) gene, the product of which participates in inorganic phosphate uptake and transport (Wang X. et al., 2014). The development of phosphorus assimilation mechanisms could have been significant in plant evolution, as increased phosphate availability in oceans is associated with the growth of larger eukaryotic organisms (Zhang S. et al., 2021).

Another feature of the gene network can be noted: the proportion of “young” genes (less than 1 million years old) was lower than their proportion in the complete genome. The “young” genes falling into this interval include 12 genes, many of which are related to immune responses to varying degrees: OsPR5 (OS01G0122000), OsNAC6 (Os01g0672100), similar to histone H4 (OS01G0835900), OsMPK3 (OS02G0148100), R2R3-MYB (OS02G0641300), R2R3-MYB (OS06G0205100), OsPR1b (OS07G0127700), histone H4 (OS07G0549900), R2R3MYB-domain protein (OS12G0564100).

The obtained data can contribute to a deeper understanding of the reconstructed gene network functioning mechanisms and serve as a basis for further selection of markers in breeding plants resistant to pathogens under elevated nitrogen fertilizer concentrations.

Search for potential marker-oriented selection targets

To search for potential marker-oriented selection targets, analysis of gene functional significance at the biological process level was conducted. Using the PANTHER resource, Gene Ontology term enrichment analysis was performed for the extended gene network. The analysis revealed 239 statistically significant biological processes (Supplementary Material 4), including key signaling pathways and responses to abiotic and biotic stresses, including fungal infections (Table 3).

**Table 3.**
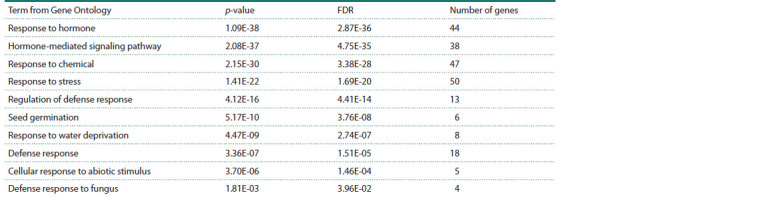
Results of biological process enrichment analysis for genes in the extended network of rice response to R. solani infection under excess nitrogen conditions Note. Analysis was performed using the PANTHER resource. The most significant biological processes related to response to various biotic and abiotic factors are presented

Although the biological process enrichment analysis provides important information about the functional significance of the gene network, the understanding of specific regulatory mechanisms is necessary for selecting effective markers. The Smart Crop knowledge base contains information about regulatory interactions between genes and biological processes, which allows identifying potential markers not only by their association with key processes but also by their regulatory potential.

**Fig. 6.**
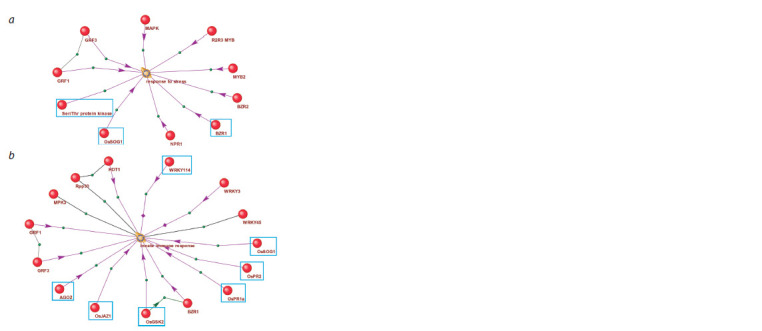
Regulation of biological processes “response to stress” (a) and “innate immune response” (b) by proteins that are components of the rice gene network response to pathogenic fungus under excess nitrogen conditions. Connections between objects marked with black lines indicate association; purple arrows indicate regulatory effects. Blue rectangles highlight proteins discussed in the text.

To search for potential markers, the gene network was supplemented with regulatory connections to biological processes using ANDVisio (Supplementary Material 5). Regulatory connections between genes and processes were classified as positive (upregulation), negative (downregulation), or without direction (regulation). Figure 6 shows regulatory networks for the processes “response to stress” and “innate immune system”, which play key roles in stress response mechanisms.

It should be noted that “response to stress” was found to be overrepresented among genes in the extended network of rice response to R. solani infection under excess nitrogen conditions (Table 3). Three proteins are important regulators of this process (Fig. 6a): BZR1 (brassinazole resistant 1), serine-threonine protein kinase SAPK4 (shown in Fig. 6a as Ser/Thr protein kinase), and transcription factor SOG1 (shown in Fig. 6a, b as OsSOG1). BZR1 is known to mediate brassinosteroid signaling by suppressing the transcription of stress response genes (Yang Y.X. et al., 2015; Cao et al., 2024). SAPK4 regulates gene expression in response to salt stress in rice (Diédhiou et al., 2008). SOG1 controls plant response to DNA damage-inducing stresses (Ogita et al., 2018; Yoshiyama et al., 2018). SOG1 is a component of the initial regulatory pathways, which allows it to be classified as a particularly important potential marker. All the considered proteins can be classified as markers controlling responses to a wide spectrum of stress effects. This characteristic makes them especially valuable for further research and potential application in plant biotechnology.

The “innate immune system” process is interesting because it is regulated by thirteen gene network participants that can be considered as promising markers associated with pathogen resistance (Fig. 6b). Key regulators of this process are proteins WRKT114 and AGO2, as well as components of the molecular genetic pathways described above (GSK, PR1, PR2, JAZ1, and SOG1). WRKT114 activates immune response during Xanthomonas oryzae pv. oryzae infection (Son et al., 2020). AGO2 regulates innate immunity through miRNA-mediated suppression of target genes during Pseudomonas syringae pv. tomato infection (Zhang X. et al., 2011). The remaining components also make significant contributions to plant immune response regulation (Song Y. et al., 2021; Johnson et al., 2023; Javed et al., 2024).

Characterization of marker genes by evolutionary age

Assessment of gene evolutionary age can provide important information for planning breeding programs, allowing prediction of specificity, functional conservation, and phenotypic effects of candidate genes. The application of gene evolutionary age analysis in experiment planning is illustrated by work on the introgression of the rice Xa21 gene. This gene provides resistance to rice bacterial blight caused by X. oryzae pv. oryzae. Xa21 was isolated from the wild species Oryza longistaminata and is an evolutionarily young gene specific to the Oryza genus. Introduction of the Xa21 gene into cultivated rice varieties led to the creation of lines with high disease resistance without negative effects on yield and grain quality (Song W.Y. et al., 1995; Wang G.L. et al., 1996).

Another example is the modification of the ERF922 gene to increase rice resistance to fungal pathogens using CRISPR/ Cas9. ERF922 is an evolutionarily young gene involved in regulating rice immune response. Its knockout led to increased resistance to rice blast without negative effects on plant growth (Wang F. et al., 2016).

Our phylostratigraphic analysis of the gene network revealed that the average evolutionary age of potential marker genes in the “innate immune response” group is 605 million years, which is significantly less than the corresponding value for the “response to stress” group (1,270 million years). These data confirm the understanding of the evolutionary youth of immune mechanisms (Han, 2019). In the “innate immune response” group, the age range extends from OsPR1a (less than 1 million years) to OsGSK2 (more than 2,101 million years), while in the “response to stress” group, from OsSOG1 (306 million years) to Ser/Thr protein kinase (1,714 million years).

It is known that genes with a greater evolutionary age participate in the functioning of more fundamental processes (Wolf et al., 2009; Domazet-Lošo, Tautz, 2010). Variations in these genes can affect multiple phenotypic traits, which may complicate selection for target properties. In this regard, evolutionarily young network genes appear most promising for marker-oriented selection: OsPR5, OsNAC6, OsMPK3, R2R3-MYB, OsPR1b, and histone H4.

## Conclusion

In this work, a systems approach incorporating a wide range of bioinformatic methods was applied to search for potential marker genes aimed at increasing rice resistance to R. solani under excess nitrogen conditions. Methods implemented in the Smart Crop cognitive system, ANDSystem, and other well-known bioinformatic resources were used. The systems analysis, implemented as a data processing pipeline, included: (1) investigation of differential gene expression; (2) reconstruction and analysis of gene networks; (3) analysis of biological process enrichment; (4) analysis of gene network evolution using phylostratigraphic analysis; (5) analysis of omics data on non-coding RNA co-expression.

Analysis of the molecular genetic interaction network connecting rice responses to excess nitrogen and R. solani infection allowed us to propose mechanisms explaining the deterioration of rice resistance to fungus under elevated nitrogen fertilizer concentrations. Three potential pathways were identified: (1) the OsGSK2-mediated pathway: OsGSK2 may be a participant in the pathway linking plant responses to excess nitrogen and R. solani fungus. At elevated levels, it can worsen plant resistance to fungus, as shown with Verticillium dahliae affecting Arabidopsis and cotton. According to our data, active OsGSK2 levels may be elevated under excess nitrogen due to decreased expression of its inhibitor (OsABI2); (2) the OsMYB44-OsWRKY6-OsPR1 pathway: all participants in this pathway are related to plant protection from biotic stresses; (3) the SOG1-Rad51-PR1/PR2 pathway: from transcription factor SOG1 through immune response gene transcription regulator Rad51 to the PR1 and PR2 genes, essential participants in pathogen response.

Reconstruction of the extended gene network allowed identification of potential markers for breeding aimed at increasing resistance to pathogens (such as R. solani) under excess nitrogen conditions. The found markers are divided into two groups: markers controlling rice responses to a wide range of stresses (7 genes) and markers modulating the immune system (11 genes).

Among the most important markers are genes that are key participants in regulatory pathways underlying the rice gene network response to R. solani pathogen under excess nitrogen conditions (OsGSK2, JAZ1, PR1/PR2, SOG1).

The obtained theoretical results can serve as a foundation for further experimental work on creating new rice varieties with increased pathogen resistance under excess nitrogen fertilizer conditions. The conducted research opens prospects for developing innovative strategies in rice breeding aimed at optimizing the balance between yield and disease resistance in modern agrotechnical conditions.

## Conflict of interest

The authors declare no conflict of interest.
